# A novel framework for evaluating the impact of individual decision-making on public health outcomes and its potential application to study antiviral treatment collection during an influenza pandemic

**DOI:** 10.1371/journal.pone.0223946

**Published:** 2019-10-17

**Authors:** Sudhir Venkatesan, Jonathan S. Nguyen-Van-Tam, Peer-Olaf Siebers

**Affiliations:** 1 Division of Epidemiology and Public Health, University of Nottingham, Nottingham, England, United Kingdom; 2 School of Computer Science, University of Nottingham, Nottingham, England, United Kingdom; Columbia University, UNITED STATES

## Abstract

The importance of accounting for social and behavioural processes when studying public health emergencies has been well-recognised. For infectious disease outbreaks in particular, several methods of incorporating individual behaviour have been put forward, but very few are based on established psychological frameworks. In this paper, we develop a decision framework based on the COM-B model of behaviour change to investigate the impact of individual decision-making on public health outcomes. We demonstrate the application of our decision framework in a proof-of-concept case study based on the 2009 A(H1N1) influenza pandemic in the UK. The National Pandemic Flu Service (NPFS) was set up in England during the pandemic as a means to provide antiviral (AV) treatment to clinically ill patients with influenza-like illness, via telephone calls or internet screening, thereby averting the need to see a doctor. The evaluated patients based on a clinical algorithm and authorised AV drugs for collection via community collection points. We applied our behavioural framework to evaluate the influence of human behaviour on AV collection rates, and subsequently to identify interventions that could help improve AV collection rates. Our model was validated against empirically collected pandemic data from 2009 in the UK. We also performed a sensitivity analysis to identify potentially effective interventions by varying model parameters. Using our behavioural framework in a proof-of-concept case study, we found that interventions geared towards increasing people’s ‘Capability’ and ‘Opportunity’ are likely to result in increased AV collection, potentially resulting in fewer influenza-related hospitalisations and deaths. We note that important behavioural data from public health emergencies are largely scarce. Insights obtained from models such as ours can, not only be very useful in designing healthcare interventions, but also inform future data collection.

## Introduction

Human behaviour is a key driver of infectious disease, and there has been a growing recognition of the importance of incorporating behaviour in epidemic models[1–3]. Over the past decade, different approaches to coupling behavioural and epidemic models have been proposed including game theoretic models to establish baseline equilibria[1], belief-based models where model agents make decisions based on *a priori* beliefs, and simple extensions to SIR (Susceptible-Infected-Recovered) models[2, 3]. In computer science and multi-agent systems communities, frameworks such as the Belief-Desire-Intention (BDI) model and layered software frameworks are extensively used to model human decision-making[4].

An effective behavioural model will allow individuals within a simulation to dynamically alter their behaviour in response to an unfolding infectious disease outbreak using decision algorithms to guide their behaviour[5, 6]. The importance of ensuring that decision algorithms are based on established sociological and psychological frameworks has been recognised[7, 8], but few attempts have been made to formalise such frameworks for use in epidemiological models [1, 2, 6, 8, 9]. Moreover, for a behavioural model to be useful to public health practitioners, it will also have to satisfy the requirement of relatively easy parametrisation of the model. One decision framework that fulfils both these criteria is the mathematical interpretation of the health belief model (HBM) as proposed by Durham and Casman[6].

According to the HBM, health-related behaviour is a result of four main constructs: perceived severity, perceived susceptibility, perceived barriers to action, and perceived benefits of the behaviour. HBM is primarily a cognitively oriented theory of behaviour change that is known to have its limitations[9, 10]. With a view to improve intervention design and healthcare policy, and to overcome limitations of previously proposed health behaviour theories, the COM-B (Capability Opportunity Motivation- Behaviour) framework was developed in 2011[11]. The COM-B framework posits that behaviour change arises from the interaction of the three essential components (C, O and M), and for an intervention to successfully bring about behaviour change, one or more of the three interacting components will have to be altered. One of the biggest advantages of the COM-B framework is that it is intervention oriented, i.e. its constructs are linked to a set of interventions and healthcare policies. The linking of theory to intervention design is in line with recommendations given by the Medical Research Council of the UK (MRC) for intervention design, and this facilitates easy evaluation of the impact of various interventions on behaviour change[12].

In this paper, we develop a simple decision framework based on the COM-B model of behaviour change. We illustrate its application in a proof-of-concept case study where we explore potential interventions that could help improve collection rates for antiviral (AV) medications during an influenza pandemic. We would like to make it clear at the outset that the goal of this paper is not to produce precise predictions about the real world. We seek to put forth a conceptual framework that can then be used to understand important drivers of health-related behaviour and gain insights into potential interventions.

## Materials and methods

### The 2009 A(H1N1) influenza pandemic and the National Pandemic Flu Service

On the 24^th^ of April 2009, the first two cases of 2009 A(H1N1) influenza pandemic in the UK were confirmed, and by the 13^th^ of June, 1000 cases had been reported[13]. In response to the rapidly increasing number of patients with confirmed pandemic influenza, the National Pandemic Flu Service (NPFS) was launched in England on 23 July 2009 to divert pressure away from primary care services for low-risk adult patients with uncomplicated influenza. The NPFS was a telephone and internet-based service that helped dispense AV medications to symptomatic patients with influenza-like illness (ILI). Patients with symptomatic ILI who had an illness duration of <7 days were issued with a unique reference code that would allow them to collect AV drugs from their nearest antiviral collection point (ACP)[13, 14]. The NPFS functioned until 11^th^ February 2010. A review of the NPFS revealed that 35.8% of the people who were authorised AV drugs did not collect them[14]. The AV drug that was dispensed through the NPFS was oseltamivir which is known to reduce the risk of hospitalisation and death in patients with influenza, especially when given within 48 hours of symptom onset [15–18]. It is conceivable that the non-collection of the medication by over one third of the AV-authorised patients could have had a notable impact on public health outcomes during the 2009 influenza pandemic in England. It is therefore important to understand drivers of health-seeking behaviour and identify interventions that will improve AV collection rates, and consequently reduce the impact of a future pandemics.

In 2015, a qualitative study was undertaken in which people were interviewed about their experience with AV medications and vaccinations during the 2009 influenza pandemic. A list of factors that are likely to influence a person’s uptake of vaccines under each component of the COM-B framework was identified[19]. We have adapted this list to inform our decision framework (Table 1). We use this list to guide the links between drivers of behaviour and behaviour change in our model.

**Table 1:** Adapted list of factors that can influence antiviral drug uptake from [18].

Capability	Opportunity	Motivation
*Psychological*: Knowledge of the disease (Awareness of disease and transmission rates) *Physical*: Not salient	*Physical*: Access to treatments *Social*: Social influences (seeing that others are being treated)	*Automatic*: Fear of infection and consequences (Physical and emotional proximity) *Reflective*: Optimistic bias

Our model’s structure is defined by two components–the decision framework and the disease states. In this section, we will describe the two components of our framework in the context of our case study.

### The decision framework

Formalising a behavioural theory involves expressing the influence of a set of variables, using the logic of a particular behavioural theory, to produce an estimate of the likelihood of a behaviour. This often means weighting constructs of a behavioural theory and combining them mathematically. A few different ways of achieving this combination of constructs have been reported in literature including additive models[8], multiplicative models[20], and logistic regression models[21]. We build on the work done by Durham and Casman[6], and use a logistic model to express our decision framework owing to its binary classification of the COM-B constructs, the ease of parametrising from public health literature and its terse representation of behaviour probability.

In the logistic expression of the COM-B model, each construct of the model (C, O and M) is classified as being in either a ‘high’ state or a ‘low’ state. The odds ratios (OR) of the behaviour when being in a particular state (‘high’ or ‘low’) are obtained either from literature or from analysing data. These values are then combined using the formula below to obtain a probability (*p*) of behaviour (*b*).

$$p(b)=\frac{{OR}_{0}*\prod OR_{i}^{x_{i}}}{1+({OR}_{0}*\prod OR_{i}^{x_{i}})},i=1,2,3$$ (1)

In Eq (1), *i* corresponds to each of the three constructs of the COM-B model–C, O and M respectively. OR_i_ represents the odds ratio of the behaviour when one of the COM-B constructs in the ‘high’ state (relative to the ‘low’ state). For example, if we assume that AV authorisations to people with ‘high’ capability (to collect AV drugs) are 3.1 times as likely to result in collections when compared to those with ‘low’ capability, the OR_1_ in Eq (1) would be 3.1. Once we have obtained OR estimates for the O and M constructs, they can be combined using Eq (1) to get a probability estimate for AV collection. OR_0_ is a constant that we will derive from fitting the model to data. It describes *p(b)* when C, O and M are in the ‘low’ state. Solving the logistic equation above will give us a value between 0 and 1. Agents with a *p(b)* value of ≥0.5 will engage in a particular behaviour (collect AV drugs).

During an ongoing pandemic, people are likely to fluctuate between states of ‘high’ and ‘low’ capability, opportunity and motivation respectively depending on the state of the disease around them and also on the behaviour of their peers. Hence, it is essential that agents in the simulation are able to update their C, O and M scores dynamically. Using the information presented in Table 1, we formulate decision rules for the updating of the COM-B constructs.

#### 1) Capability

Using the COM-B behaviour model, Rubinstein et al. (2015) identified that a person’s capability to engage in the thought process necessary to enact a particular behaviour (collect AV drugs) is influenced by awareness of the disease rates of the ongoing outbreak[19]. The capability of a person to make a particular decision is more likely to be influenced by local disease activity in their immediate networks, rather than global-level prevalence estimates. Indeed, the importance of local awareness during an outbreak has been reported before[22]. Even within local networks, disease activity is a combination of new infections occurring each day, and the total number (cumulative) of infections that have occurred in a local network over the course of the outbreak. Therefore, in the context of our case study, an agent’s capability to collect authorised AV drugs would depend on its level of awareness of local disease activity, both incident cases and prevalent cases. However, it is unlikely all individuals would actively seek out information on disease rates during a pandemic. Further, there is evidence to suggest the possibility of a cognitive discounting of earlier hazards in favour of more recent events[23].

In our decision framework, we propose to incorporate the effects of awareness of disease activity using both the cumulative as well as daily incidence of influenza cases. We model the biases that can affect an agent’s perception of local disease rates by using a weighted discount rate (*δ*) which can take any value between 0 and 1 (Eq 2).

$$c=\sum_{i=0}^{t-2}\delta^{i}*I_{t-i-1}$$ (2)

Capability *C* is represented as the weighted sum of the number of new influenza cases *I* at time *t*, discounted at the rate δ. This approach to calculating discounted cumulative incidence is consistent with previous behaviour models[6, 8]. Once an agent has determined its Capability score, it then compares it with a threshold α to determine if it is in the ‘high’ or ‘low’ state of Capability (Eq 3).

$$C=\left\{ \begin{array}{l} high,\;c>\alpha \\ low,\;else \end{array}\right.$$ (3)

### 2) Opportunity

According to the COM-B model, the opportunity to engage in a particular behaviour represents all of the factors that influence the behaviour but are outside the control of the individual[11]. Rubinstein et al. (2015) identified ‘access to treatments’ and ‘seeing other being treated’ as affecting an individual’s opportunity to engage in behaviour change (Table 1). An ecological study performed in the UK also reported that the likelihood of AV collection significantly reduced with increasing distance to an ACP[24]. For our case study, we model ‘Opportunity’ as a function of the distance to an ACP and seeing others in their community who have collected AVs (*C*_*n*_), with the distance to an ACP having an inverse association with ‘Opportunity’ (i.e. a decreasing likelihood of AV collection with an increasing distance to an ACP). Agents in our simulation then compare their respective ‘Opportunity’ scores (*O*) to a threshold *η* to determine if they are in a state of ‘low’ or ‘high’ opportunity (Eq 4). There is also evidence for the association between socioeconomic depravation (SED) and access to influenza treatment [24, 25]. We do not explicitly include this in our current study due to the lack of available data. However, a measure of SED, such as the English Index of Multiple depravation (IMD) [26], could easily be incorporated into the Opportunity score as a multiplicative factor.

$$O=\left\{ \begin{array}{l} high,\;(\frac{C_{n}}{Distance})>\eta \\ low,\;else \end{array}\right.$$ (4)

#### 3) Motivation

‘Motivation’ includes the mental processes that direct behaviour beyond conscious decision-making[11]. According to the COM-B model, ‘motivation’ is comprised of an automatic and a reflective component. In the study by Rubinstein et al. (2015), fear from proximity (physical and emotional) to the pandemic, and beliefs about consequences of being ill from pandemic influenza was identified as the automatic component of motivation that influenced people’s decision to take AV drugs (Table 1). The belief amongst some people that, because they followed an active lifestyle, they would make an easy recovery from pandemic influenza without needing to take AV treatment was found to be the reflective component of motivation[19]. In our decision framework, we used the case hospitalisation risk (CHR) as an indicator of physical and emotional proximity to severe outcomes from the disease, and we will use a variable, *OB*, to account for optimistic bias. *OB* can take any value between 0 and 1 with higher values indicating an increased optimistic bias. Each agent’s motivation (*M*) will be evaluated against a threshold *θ* to determine if they were associated with a ‘high’ or ‘low’ state of motivation (Eq 5).

$$M=\left\{ \begin{array}{l} high,\;(\frac{No.hosptialisations}{No.illnesses})/OB>\theta \\ low,\;else \end{array}\right.$$ (5)

### The epidemic model

We use a simple epidemic ABM to simulate a hypothetical influenza pandemic during which agents make a decision to collect authorised AV drugs or not. At any given point during the simulation, an individual agent is in one of four disease states–Susceptible, Exposed, Infected, or Recovered. An infected agent would, in turn, infect a susceptible agent in its local network, and transitions between disease states were determined by specified transmission parameters. For our case study, we assume that only agents who have been infected (i.e. in the ‘Infected’ state) can be authorised AV drugs. Once an infected agent has been authorised AV drugs, the decision framework described in the previous section is triggered, and the agent then makes the decision to collect the authorised AV, or not, based on its capability, opportunity and motivation at that particular point in time.

### Model parametrisation and experimentation

Using the model structure described above, we simulated 10,000 agents and one ACP, keeping with the population-to-ACP ratio that was seen in England during the 2009 A(H1N1) influenza pandemic[14]. To simulate neighbourhoods within a town or a city, we implemented a spatially structured model where the agent space was divided in to nine equally sized regions and, at start-up, agents were randomly assigned to one of the nine regions. A distance-base network was used, and two agents were connected if the distance between them was lesser than a defined maximum distance. Epidemic parameters relate mainly to the 2009 influenza pandemic, and most were chosen from literature (Table 2); the probability of transmission was calibrated to match the duration of the second peak of the 2009 influenza pandemic in the UK. We assumed that AV treatment did not have an impact on transmission. Once an agent entered the ‘Infected’ state, an NPFS call would be made, and based on data from the 2009, 75% of NPFS calls would result in an AV authorisation[27]. Once an AV authorisation was made to an infected agent, the decision module is triggered and the agent then makes the decision to collect treatment based on the decision rules described in earlier section of this paper using information from their respective local environments.

**Table 2:** Input parameters for the agent-based model.

Parameter	Value *Probability distribution*	Source
Contact rate, Mean (SD)	11.74 (7.67) *Normal*	[28]
Duration of illness, Median (IQR) in days	6.5 (5 to 8) *Triangular*	[29]
Probability of infection, Range	0.015 to 0.020 *Uniform*	Calibrated
Latency period, Mean (SD) in days	1.6 (0.26) *Normal*	[30]
Case hospitalisation risk	0.2%	[31]
Likelihood of AV collection for ‘high’ Capability vs. ‘Low’, OR (95% CI)	2.80 (2.52 to 3.08) *Triangular*	[32]
Likelihood of AV collection for ‘high’ Motivation vs. ‘Low’, OR (95% CI)	2.71 (2.08 to 3.53) *Triangular*	[33]
Likelihood of AV ‘high’ Opportunity vs. ‘Low’, OR (95% CI)	4 (3 to 5) *Triangular*	Assumed based on [24]

SD: Standard deviation; IQR: Interquartile range; OR: Odds ratio; CI: Confidence Intervals

Given that several behavioural parameters were unavailable from literature, we specified four parameters as calibration parameters–the three behavioural thresholds (α, η and θ), and the probability of AV collection when C, O and M are in the ‘low’ state (OR_0_ from Eq 1). Given the high dimensionality of the model parameters, an exhaustive search of the parameter space was not feasible. Therefore, we followed a three-step model calibration method based on the Werker-Brenner calibration approach[34, 35]. We first used existing empirical data for parameters, where they were available. For the four behavioural parameters where no data were available, we specified wide ranges after investigating preliminary model runs. We then performed 1,000 model realisations to narrow down parameter ranges by comparing the model output to empirical data and using an 80% confidence limit (error percent: 0.5%); parameter sets that produced output that fell outside this range were discarded. We then used subjective judgement to refine the obtained parameter ranges (abduction) and then performed another set of 1,000 calibration runs to further narrow the plausible parameter space. For our calibration, we used empirical time series data from the 2009 A(H1N1) influenza pandemic on the weekly proportion of NPFS-based AV authorisations that resulted in collections[31]. Given that the NPFS was unavailable for the first half of the first peak of the 2009 influenza pandemic, we calibrated our model to the second wave of the pandemic (from 31 August 2009 to 11 February 2010–23 weeks and 4 days) to ensure that we had data on an entire epidemic curve.

Model parameters were varied over a probability distribution to induce agent heterogeneity (Tables 2 and 3). We performed a 5,000 iteration Monte Carlo uncertainty analysis by simultaneously varying all model parameters over the identified plausible ranges to analyse the impact of heterogeneity on our model output. Finally, we performed a set of sensitivity analyses by examining the model output when α, η and θ were varied by 50%, and when *δ* was varied from 0 to 1 in increments of 0.1. For the threshold variables, we also looked at a hypothetical scenario where α, η and θ were set at 0 (i.e. there were no barriers to capability, opportunity, or motivation).

**Table 3:** Final calibration parameter ranges.

Parameter	Range
α	183 to 185
η	0.15 to 0.20
θ	0.20 to 0.25
OR_0_	0.1 to 0.3

## Results

**Fig 1:**
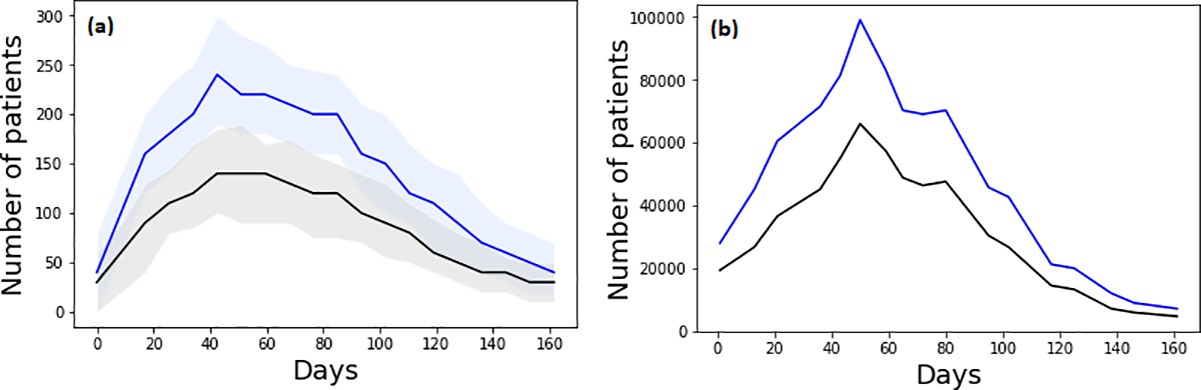
A comparison of AV treatment collection rates relative to ILI consultations (based on NPFS authorisations) between the model output and empirical data from 2009. For the model output, the solid lines represent the mean model outputs and the shaded regions represent the range of the middle 80% of model output over 1,000 iterations.

Using our COM-B decision framework, our model was able to approximate the dynamics of the number of AV collections relative to the NPFS-related ILI consultations (Fig 1). The peak of the pandemic was observed on day 50 in the empirical data, whereas our model predicted that it would occur on day 43. A second smaller peak that occurred on day 80 was consistent our model output. Although the absolute numbers between the model output and the empirical data are not directly comparable, the relative differences and the dynamics between them were similar. Between day 40 and day 100 of the outbreak, the mean model output (over 1,000 iterations) suggested that between 58 and 63% of ILI consultations (that resulted in NPFS authorisations) led to AV collections. This proportion for the corresponding time period in the empirical data was observed to be slightly higher at 62 to 68%. Our final calibration parameter ranges are presented in Table 3.

**Fig 2:**
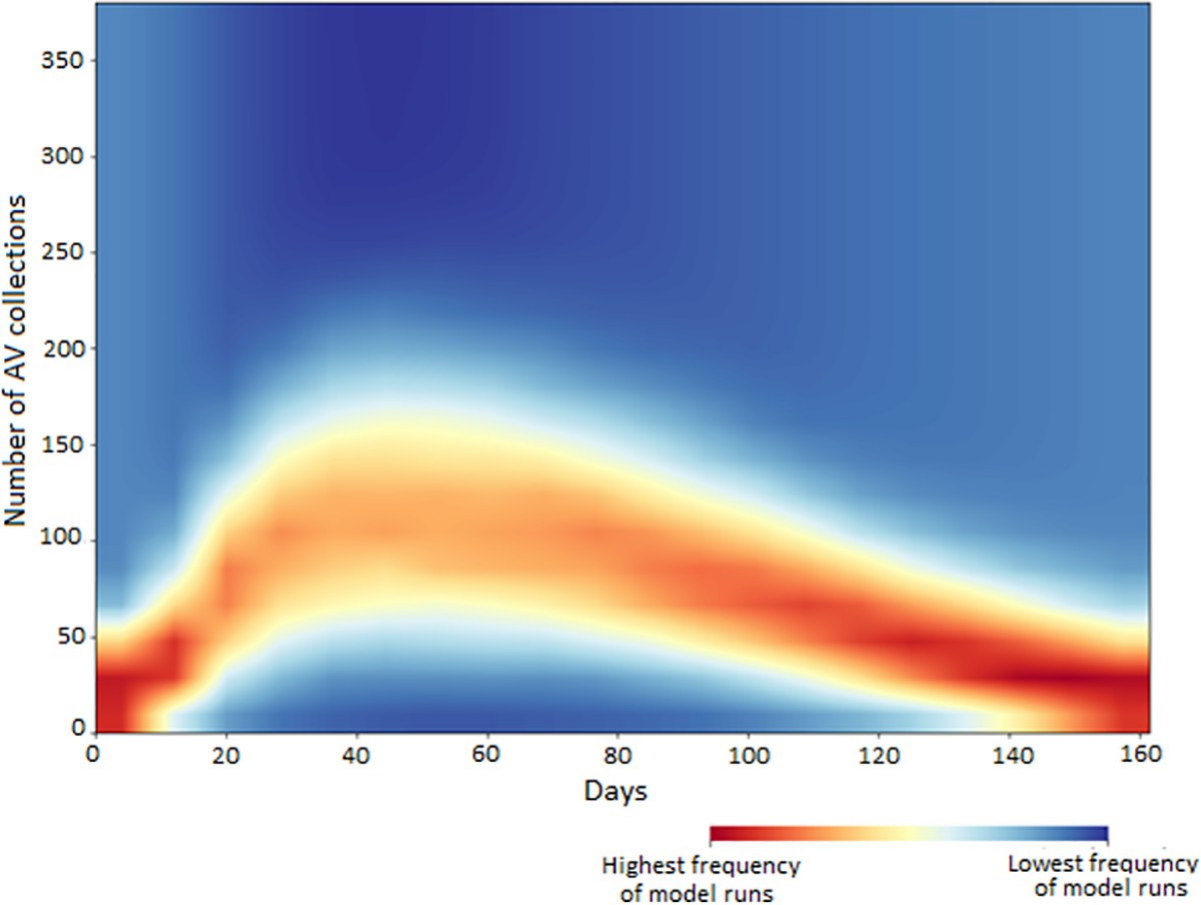
A 2D histogram showing output from a 5,000 iteration Monte Carlo uncertainty analysis.

Results from our Monte Carlo analysis where we varied all parameters across specified ranges over 5,000 random seeds are presented in Fig 2.

**Fig 3:**
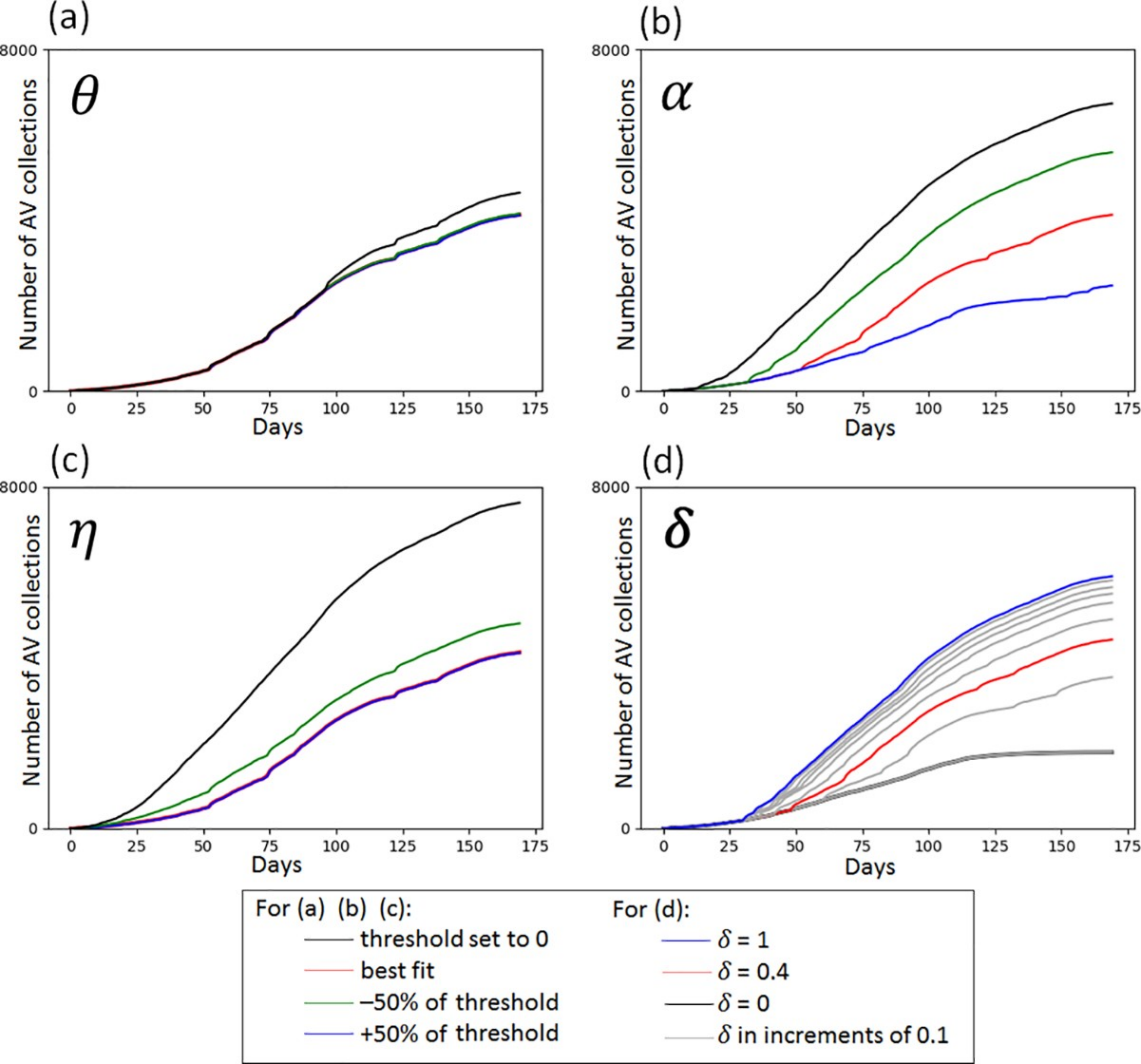
Sensitivity analyses: Impact of varying α, η, θ and *δ* on AV collection.

Our sensitivity analyses showed that the capability threshold, *α*, had the biggest impact on the model output. Reducing the *α* value by 50% of the best-fit value increased AV collection by almost 36%, and a 50% increase in the best-fit value of *α* was associated with a reduction in AV collections by about 40%. Similar associations were found with varying the discount rate *δ*, by 50%. Decreasing the best-fit value for the opportunity threshold *η* was seen to be associated with a 16% increase in antiviral collections. However, an increase in the best-fit *η* by 50% did not make a significant difference to AV collections. Varying the motivation threshold was also not seen to have an impact on total AV collections. Setting the α, η and θ threshold values at 0 was associated with significant increases in AV collection (Fig 3).

## Discussion

The main contribution of this paper is the development of a novel decision framework, based on an empirically identified set of constructs [19]. Our decision framework is novel in that, to the best of our knowledge, it is the first implementation of the COM-B model of behaviour change in an epidemic ABM. Our behavioural-epidemic model seeks to use the COM-B model to demonstrate how incorporating the elements of capability, opportunity, and motivation into epidemic models can generate important insights into how interventions could be developed to improve AV collection rates (and therefore treatment rates) for future pandemics. The proof-of-concept implementation of our decision framework is based on the UK National Pandemic Flu Service which operated in 2009 and sought to divert pressure away from primary care services. Using this approach 1.2 million treatment courses of antivirals drugs were dispensed[36]. It is intended that this system will be used again in the future[37], and other countries are now considering similar models[38].

Our model slightly underestimated the proportion collecting authorised AV drugs in the middle part of the outbreak when compared to data observed from 2009. Although, the dynamics, for the most part, were consistent with empirical data. The sensitivity of the model to the capability threshold *α* suggests that a person’s awareness of an ongoing epidemic can greatly influence how capable they are of making healthcare decisions. The discounting rate *δ* was also seen to have had a significant impact on AV collection rate. This is not surprising as the capability threshold *α* is a function of *δ*. The model is not sensitive to changes in the motivation threshold θ is because we have assumed a constant CHR in our model.

The benefit of using the COM-B model is that capability, opportunity and motivation have already been mapped to a set of interventions that are likely to influence that particular construct[11]. High threshold values for α, η and θ can be seen as barriers to AV collection, and low threshold values as facilitators. An educational media campaign during an ongoing pandemic could contribute to an increase in a person’s awareness of the pandemic (thereby increasing capability) and simultaneously also reduce optimistic bias (increasing motivation). We model optimistic bias by randomly assigning levels of this bias across the population. This could be improved in future work by first identifying specific population groups that are likely to have increased levels of optimistic bias and then accounting for this in the ABM. Increasing the number of ACPs (environmental restructuring) could reduce the opportunity threshold (by reducing the distance to the ACP, improving access to treatment), and also improve motivation. Findings from our exploratory analysis reinforce the findings of the qualitative study by Rubinstein et al. that effective interventions to increase AV uptake are likely to be multidimensional[19]. In deciding the most suitable intervention to increase AV collection rates, the government will need to consider the cost-effectiveness of each potential intervention.

The structure of our decision framework is rooted in the work done by Durham and Casman[6], however, our framework differs from theirs in a number of important ways. Agents in our simulation make decisions based on information from their local networks, unlike the global approach followed by Durham and Casman, and we do not de-couple the epidemic and the behavioural components of the model as has been done in some previous work[6, 39]. But most importantly, what our framework adds to existing evidence is, the drivers of C, O and M that influence behaviour are based on an empirically validated framework that has already been linked to policy, whereas the main limitation of decision frameworks based on the HBM is that links between potential drivers of behaviour change and health beliefs are poorly understood[6]. Another behaviour model based on the HBM used parameters such as attitude, norms, and worry to obtain a behaviour score; however, the authors note that their model output fit poorly with empirical data[8]. The difficulty in validating and fitting behaviour models to empirical data has been noted by others as well [40, 41]. A systematic review of published studies reported that only 15% of studies involving behaviour change models in epidemiology performed any type of validation with empirical data [3]. This systematic review further noted that most behavioural models used were theoretical models that were developed independently from empirical data. We have followed a more practical approach and attempted to validate our simulation model. Validation of a simulation model requires validation of the underlying conceptual model as well as the simulation output [42]. The conceptual model that we have used is based the COM-B model of behaviour change that is widely used within public health and is recommended by leading research organisations [12]. We validate our simulation output against NPFS data from the 2009 influenza pandemic in the UK, noting that the dynamics observed in our simulation output was similar to what was observed in 2009.

One important consequence of a lack of suitable behaviour data is that multiple model parameters may have to be calibrated to ensure that model output is consistent with empirical data. Previous behaviour models have calibrated up to six input parameters [6, 8]. We have tried to minimise the number of parameters calibrated by only calibrating the four essential behavioural parameters. However, we varied the calibrated parameters over a range in a set of sensitivity analyses to evaluate their impact on the model output. This is one way of validating a model with calibrated parameters [42, 43]. Our paper also demonstrates one of the early epidemiological applications of the Werker-Brenner calibration approach [34, 35], which is particularly well-suited to using existing knowledge in guiding calibration of parameters with wide uncertainty. The sensitivity analyses produced results that were qualitatively and quantitatively consistent with what would be expected empirically (i.e. a reduction in behavioural thresholds improved the likelihood of behaviour change). Nevertheless, our exploratory study could be used to guide collection of behavioural data in the future. OR_0_ can be easily estimated by analysing public health survey data. The three behavioural thresholds can be estimated statistically from behavioural data collected over a period of time.

This study is not without limitations or simplifying assumptions. We assumed that AV treatment was not used for prophylaxis and was only given to patients with clinical illness. This is in line with what was observed during the treatment-only phase of the pandemic in 2009 during which the NPFS operated[31]. We also assumed that AV treatment does not have an impact on transmission. There is evidence to suggest that the population-level impact of AV treatment on transmission is likely to be quite small[44]. We do not account for varying contact rates by agent location (workplace, school, or home), the impact of other pandemic influenza mitigation measures, or the impact of transport disruptions. All modelling endeavours require a compromise between striving for an accurate representation of reality and computational feasibility. We have deliberately implemented a relatively simple epidemic model on a small population, as the network effects in our decision framework make modelling larger populations computationally expensive.

We fully recognise that our decision framework is not a perfect representation of the COM-B behaviour change model, and indeed, the COM-B behaviour change model may not be a perfect representation of behaviour change. We base the COM-B constructs for our proof-of-concept implementation on the empirically identified set of factors by Rubinstein et al., but we are aware that there may be other factors driving healthcare-seeking behaviour that we have not accounted for in our model. Influences such as ‘fear’ are not straightforward to incorporate into an ABM, but we have used what we think are sensible proxies to model the influence of factors that are hard to represent (such as fear) in a dynamic model. We would again like to stress that our current study is not suited to make forecasts, rather, it is intended to serve as a tool to help explore the impact of various factors potentially driving healthcare behaviour in a dynamic model.

## Conclusions

Our decision framework demonstrates how the COM-B behaviour change model can be incorporated into an ABM to study the effectiveness of healthcare interventions. This is, to the best of our knowledge, the first attempt at formalising the COM-B model for use in an ABM. Our decision framework lends itself quite well to easy parameterisation from surveys or other public health data. We recommend that for future applications of our framework, the associations between individual factors driving behaviour change and the COM-B constructs first be empirically identified through a qualitative study, and then the simulation model ideally be informed by behaviour data collected specifically for the case study of interest. Capability, opportunity and motivation are also likely to dynamically influence each other. For example, increasing a person’s awareness of a disease (capability) could also influence their optimistic bias (motivation). Future applications of our framework could explore the dynamic interactions between the COM-B constructs. Finally, we have assumed a crisp implementation of behaviour probability (behaviour threshold of ≥50% probability). In reality, however, decisions are frequently made under conditions of uncertainty and imprecision. Using fuzzy decision rules [45] to guide agent behaviour could be a direction for future research to improve upon our current framework.

## Supporting information

S1 File The model described in the manuscript has been provided as a set of Java files in the supporting information. (RAR)
